# Validity and Reliability of the Daily Activity Behaviours Questionnaire (DABQ) for Assessment of Time Spent in Sleep, Sedentary Behaviour, and Physical Activity

**DOI:** 10.3390/ijerph19095362

**Published:** 2022-04-28

**Authors:** Kaja Kastelic, Nejc Šarabon, Michael D. Burnard, Željko Pedišić

**Affiliations:** 1 Andrej Marušič Institute, University of Primorska, 6000 Koper, Slovenia; kaja.kastelic@iam.upr.si (K.K.); mike.burnard@innorenew.eu (M.D.B.); 2 InnoRenew CoE, 6310 Izola, Slovenia; nejc.sarabon@fvz.upr.si; 3 Faculty of Health Sciences, University of Primorska, 6310 Izola, Slovenia; 4 Institute for Health and Sport, Victoria University, Melbourne 8001, Australia

**Keywords:** time-use questionnaire, time-use composition, 24-h movement behaviours, physical behaviours, time-use epidemiology

## Abstract

Sleep, sedentary behaviour (SB), and physical activity are among key behavioural determinants of health. There is a need to evaluate questionnaires that capture movement behaviours across the full 24-h day. The aim of this study was to examine the measurement properties of the Daily Activity Behaviours Questionnaire (DABQ), a novel questionnaire (with a past seven-day recall period) for estimating the time spent in sleep, SB, light physical activity (LPA), and moderate-to-vigorous physical activity (MVPA) among adults. A sample of 126 adults was recruited. DABQ was administered to the participants on two occasions seven days apart to examine its test-retest reliability. The convergent validity of DABQ estimates was explored against activPAL4 accelerometer/inclinometer estimates. Intraclass correlation coefficients for absolute agreement and consistency between the times spent in sleep, SB, LPA, and MVPA estimated by DABQ in the test and re-test ranged from 0.59 to 0.69. Spearman’s correlations between the times spent in sleep, SB, LPA, and MVPA estimated by DABQ and activPAL4 ranged from 0.38 to 0.66. In terms of reliability and validity, DABQ is comparable with existing questionnaires; however, it has an important advantage of enabling a comprehensive assessment of all four 24-h movement behaviours. The measurement properties of DABQ make it suitable for large-scale epidemiological studies on 24-h movement behaviours.

## 1. Introduction

Sleep, sedentary behaviour (SB), and physical activity are among key behavioural determinants of health [1,2]. Studies suggest that more time spent in moderate-to-vigorous physical activity (MVPA) and adequate sleep duration (e.g., seven to nine hours per day for adults [3]) are associated with a reduced risk of major chronic diseases, such as cardiovascular disease, type II diabetes, and some types of cancer [1,2]. Studies have also suggested that more time spent in SB is associated with adverse health outcomes [4] while light physical activity (LPA; i.e., physical activity characterised by low energy expenditure of 1.5 to 3.0 METs) is beneficial for health [5,6].

Given that the time spent in SB, LPA, and MVPA is equal to total waking time, the daily durations of these movement behaviours are co-dependent with sleep duration. A recognition that sleep, SB, LPA, and MVPA are all associated with health and that their durations in any given period (e.g., day, week) are co-dependent led to a recent change in research paradigms towards *time-use epidemiology* [3,7,8]. This rapidly emerging research field acknowledges that sleep, SB, LPA, and MVPA collectively impact health and that they should be studied in combination. The times spent in sleep, SB, LPA, and MVPA are now considered inseparable parts of a time-use composition, and because their sum always equals to 24 h/day, they are commonly referred to as “24-h movement behaviours”.

In epidemiological studies, assessment of sleep, SB, LPA, or MVPA is usually performed using self-reports, such as questionnaires and 24-h recalls, or devices, such as accelerometers. While device-based measures generally have somewhat higher validity and reliability, self-reports may have advantage in terms of lower burden for participants and researchers, comprehensiveness, lower cost, and sustainability [9,10,11,12]. The price of measures used for health surveillance and monitoring is particularly important in low- and middle-income countries [12]. Therefore, despite some advantages of device-based measures of sleep, SB, LPA, and MVPA, self-reports remain the preferred measures in health surveillance systems [11,13,14]. Accordingly, most epidemiological evidence on sleep, SB, and physical activity comes from studies that collected data using questionnaires [1,2,4].

Given that, until recently, epidemiological studies have usually focused on one of the movement behaviours in isolation, most existing questionnaires were developed to assess either sleep, SB, or physical activity individually. Additionally, most physical activity questionnaires assess only MVPA and do not include questions on LPA. Some questionnaires, such as the International Physical Activity Questionnaire (IPAQ) [15] and the Global Physical Activity Questionnaire (GPAQ) [16], provide insight into two movement behaviours (i.e., MVPA and SB). However, none of the previous studies on 24-h movement behaviours identified by three recent systematic reviews [8,17,18] used a validated questionnaire that was developed specifically to assess the full time-use composition, including sleep, SB, LPA, and MVPA. The main reason for this is likely a lack of such questionnaires.

Following the *Viable Integrative Research in Time-Use Epidemiology* (VIRTUE) framework (that encompasses research methods for time-use epidemiology, time-use interventions, and prevalence, determinants, and outcomes of (un)healthy use of time), and to support the development of time-use epidemiology research and population health surveillance, there is a need for a validated questionnaire that captures movement behaviours across the full 24-h day [19]. Therefore, the aim of this study was to examine the measurement properties of a novel questionnaire for estimating the amounts of time spent in sleep, SB, LPA, and MVPA among adults. We hypothesized that all estimates from the novel questionnaire would show satisfactory test-retest reliability and convergent validity.

## 2. Materials and Methods

### 2.1. Participants

Adults with diverse working backgrounds were invited to participate in the study, to capture various sleep, SB, LPA, and MVPA patterns. This included employees of a university, members of the police force, and employees of a national gas enterprise. We aimed to recruit a sex-balanced sample of at least 100 participants. This minimum sample size was targeted to ensure a relatively narrow width (±0.10) of the 95% confidence interval (CI) for an intraclass correlation coefficient (ICC) of 0.70, according to Bonnet’s formula [20]. The inclusion criteria were aged 18–65 years, and able to walk independently. The final sample included 126 participants. All participants signed an informed consent before engaging in the study. This study was performed in accordance with the Declaration of Helsinki, and it was approved by the Republic of Slovenia National Medical Ethics Committee (approval number: 0120–557/2017/4).

### 2.2. Measures

#### 2.2.1. Sleep, Sedentary Behaviour, and Physical Activity

Participants were asked to complete a web-based Daily Activity Behaviours Questionnaire (DABQ) on the day they entered the study (test) and after seven days (re-test). The DABQ is a novel 32-item questionnaire that asks about sleep and domain-specific (including occupational, commuting, and other non-occupational) SB and physical activity in the past seven days. The questionnaire, user manual, and Microsoft Excel spreadsheet developed specifically for DABQ data processing can be found at www.healthytimeuse.com/en/pages/7 (accessed on 20 April 2022). The questionnaire is available in Croatian, English, German, and Slovenian languages. For the purpose of this study, we used the Slovenian version of the questionnaire. DABQ can be used to assess: (1) the total amounts of time spent in sleep, SB, LPA, and MVPA within a 24-h period; (2) several indicators of sleep quality, including sleep latency, wake after sleep onset, and napping frequency and duration; and (3) domain-specific (i.e., occupational, commuting, and other non-occupational) SB and physical activity. Responses to some questions are provided as time (hh:mm); for example: “*In the past seven days, what time did you on average arrive at your workplace?*” Responses to the questions on SB are provided using a visual analogue scale (ranging from “*none of the time*” to “*all of the time*”) as suggested previously [21]; for example: “*In the past seven days, what proportion of your time at the workplace did you spend sitting?*” Responses to some questions are provided on the *yes/no* scale; for example: “*In the past seven days, did your work duties include any physically more demanding tasks?*” Responses to most questions are provided as numerical entries (i.e., number of days, hours, and/or minutes); for example: *“How many days did you engage in the above mentioned physically more demanding tasks in the past seven days?”*

A researcher placed an activPAL4 micro (PAL Technologies Ltd., Glasgow, Scotland) device on the anterior aspect of the participant’s right thigh, midway between the anterior superior iliac spine and the knee, using an adhesive dressing (Tegaderm). ActivPAL4 is a thigh-worn accelerometer primarily developed to differentiate between lying/sitting, standing, and stepping [22]. It can also provide estimates of SB, LPA, and MVPA [23]. Data collected with a 24-h wear protocol also support estimations of sleep duration [24]. Participants were instructed to wear activPAL4 continuously for a full seven-day period (except while swimming). For activPAL4 initialization and data collection, proprietary software (PALconnect version 8.11.4.89, PAL Technologies Ltd., Glasgow, Scotland) was used, and the activPAL4 default recording mode was chosen (10 bit resolution, 20 Hz sampling frequency, ±4 g range of acceleration). Participants with at least five valid days of activPAL4 data were included in the analysis [25], where a valid day was considered if the participant wore activPAL4 for more than 20 h/day.

A sleep diary was used to collect information about the time when participants intended to fall asleep and when they woke up and about the duration of daytime napping. Participants were asked to complete a sleep diary on a daily basis for the seven days that corresponded with activPAL4 measurements.

#### 2.2.2. Sociodemographic Data

Information on sex (male/female), age (years), body height (cm), body weight (kg), educational level (primary school/secondary school/college/university), and work schedule (non-shift work/shift work) were self-reported by participants during the first meeting with the researcher. Body mass index (BMI) was additionally calculated as body weight divided by squared body height (kg/m^2^).

### 2.3. Data Processing

The raw data collected using DABQ were processed using a Microsoft Excel spreadsheet developed specifically for this purpose. Sleep duration was calculated based on the self-reported time in bed, while taking into account sleep latency, wake after sleep onset, and daytime napping. The time spent in SB was calculated as a sum of self-reported occupational SB, commuting SB, and other non-occupational SB. The time spent in MVPA was calculated as a sum of self-reported occupational MVPA, commuting MVPA, and other non-occupational MVPA. All the remaining time in a 24-h day was considered as the time spent in LPA (including occupational LPA, commuting LPA, and other non-occupational LPA).

The data collected by activPAL4s were processed using the CREA algorithm (PALanalysis, version 8.11.4.61, PAL Technologies Ltd., Glasgow, Scotland). A 24-h protocol that allows for up to four hours of non-wear time was applied. The CREA algorithm detects the onset (hh:mm) and offset (hh:mm) of time in bed by identifying the longest sitting/lying event each day (allowing for bathroom breaks/interruptions of ≤15 min in total) [26]. This algorithm was shown to correctly identify 92% of the self-reported time in bed [24]. Since time in bed is somewhat longer than sleep time, and to reduce the measurement error associated with sleep diaries and CREA algorithm, it was recently proposed to combine the two methods when estimating sleep time [24]. For sleep onset, diary information was used if there were no bursts of upright events recorded by activPAL4 within the following 20-min period. Otherwise, the sleep onset was set at the first sitting/lying event recorded by activPAL4 after the burst of upright events. For the sleep offset, diary information was used if it preceded the first burst of upright events recorded by activPAL4 after more than one hour of sitting/lying events. Otherwise, the SLP offset was set at the last sitting/lying event before the burst of upright events recorded by activPAL4. A similar approach was used in previous studies [27,28]. Napping duration from the sleep diary was added to sleep time to calculate total sleep time. The napping duration from the sleep diary was also deducted from the SB duration estimated by activPAL4. “Events” files were further processed in R studio using the package *activpalProcessing* [29] to obtain estimates of MVPA. LPA was calculated as a difference between 24-h and the sum of sleep, SB, MVPA, and non-wear time. Non-wear time was then proportionally reallocated to wake-time movement behaviours only [30]. Finally, the average amounts of time spent in sleep, SB, LPA, and MVPA per day were calculated as their arithmetic means across valid days.

### 2.4. Statistical Analysis

The data were analysed in R (version 4.0.5, R Foundation for Statistical Computing, Vienna, Austria) [31] and R Studio 1.4.1106 [32] using the packages *BlandAltmanLeh* [33], *DescTools* [34], *dplyr* [35], *ggplot2* [36], *psych* [37], and *stats* [38]. Sample characteristics were presented using absolute and relative (%) frequencies. The amounts of time spent in sleep, SB, LPA, and MVPA estimated using DABQ and activPAL4 were presented as means and standard deviations (SDs). The test-retest reliability of DABQ was explored by comparing estimates obtained from the two DABQ measurements. The convergent validity of DABQ estimates was explored by comparing them with the activPAL4 estimates. Further, two-way mixed model ICCs and their 95% CIs were calculated to explore the extent of absolute agreement (ICC[A,1]) and consistency (ICC[C,1]) between the DABQ estimates in the test and re-test and between DABQ and activPAL4 estimates. Spearman’s *ρ* and its 95% bootstrap CI were calculated as a measure of rank-order correlation between the DABQ estimates in the test and re-test and between DABQ and activPAL4 estimates.

To further explore the convergent validity, Bland–Altman plots were constructed. The mean difference (i.e., difference between arithmetic means of DABQ and activPAL4 estimates) and its 95% CI were calculated to explore the accuracy (i.e., systematic difference) of the DABQ estimates, when compared with the activPAL4 estimates. The limits of agreement (i.e., ±1.96 × SD of differences between DABQ and activPAL4 estimates) and their 95% CIs were calculated to explore the precision (i.e., random differences) of the DABQ estimates, when compared with the activPAL4 estimates.

## 3. Results

### 3.1. Participant Characteristics

A total of 114 participants completed DABQ on both occasions, and they were therefore included in the analysis of test-retest reliability. A total of 107 participants completed DABQ on the second occasion and had at least five valid days of activPAL4 data, and they were therefore included in the analysis of convergent validity. Ninety-eight participants provided seven valid days of activPAL4 data, seven participants provided six valid days, and two participants provided five valid days.

The mean (±SD) age of the participants was 39 ± 8 years, with a range from 24 to 56 years. Approximately 40 percent of participants were females, and nearly half of the participants had “normal” BMI (Table 1). Most participants reported that they work five days per week, with a mean of approximately 41 working hours per week. Around half of the participants had a university degree and reported having a shift-work schedule.

### 3.2. Reliability of the Daily Activity Behaviours Questionnaire (DABQ)

The absolute agreement and consistency between the amounts of time spent in sleep, SB, LPA, and MVPA estimated by DABQ in the test and re-test were in the range from 0.59 and 0.69 (Table 2). Spearman’s correlations between the amounts of time spent in sleep, SB, LPA, and MVPA estimated by DABQ in the test and re-test ranged from 0.60 to 0.67. The mean test-retest differences (95% CI) in the amounts of time spent in sleep, SB, LPA, and MVPA estimated by DABQ were −13 min/day (95% CI: −23, −3), 0 min/day (95% CI: −25, 26), 7 min/day (95% CI: −17, 31), and 6 min/day (95% CI: 0, 12), respectively. The test-retest reliability for most other measures obtained from DABQ was similarly high (Table S1).

### 3.3. Validity of the Daily Activity Behaviours Questionnaire (DABQ)

Both the absolute agreement and consistency between DABQ and activPAL4 estimates of sleep duration were 0.63 (95% CI: 0.52, 0.71) (Table 3). For SB, LPA, and MVPA, we found somewhat lower absolute agreement (ICC[A,1] = 0.22 to 0.29) and consistency (ICC[C,1] = 0.35 to 0.47) between DABQ and activPAL4 estimates. Spearman’s correlations between the amounts of time spent in sleep, SB, LPA, and MVPA estimated by DABQ and activPAL4 ranged from 0.38 to 0.66.

**Figure 1:**
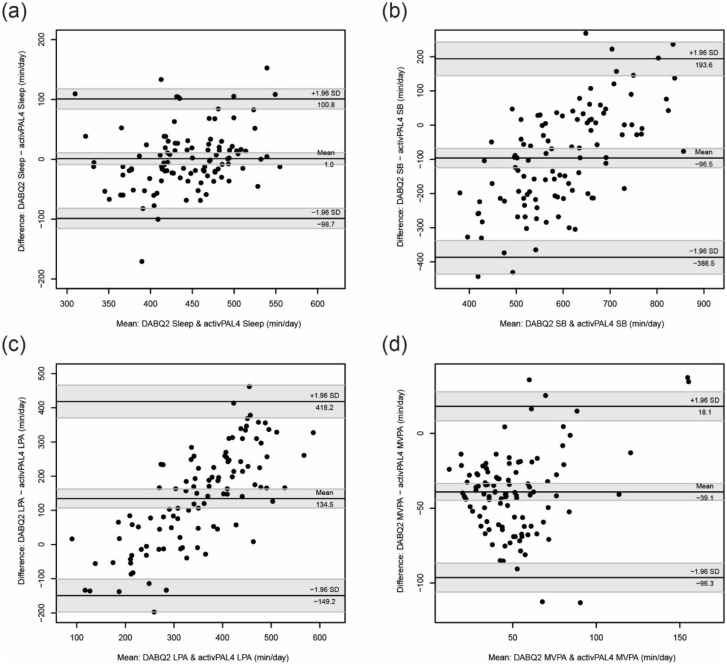
Bland–Altman plots comparing DABQ2 and activPAL4 estimates of (**a**) sleep duration, (**b**) time spent in SB, (**c**) time spent in LPA, and (**d**) time spent in MVPA. Lines show the mean difference and limits of agreement while shaded areas show their 95% confidence intervals. DABQ2, Daily Activity Behaviours Questionnaire completed on the second occasion; activPAL4, activPAL4 accelerometer/inclinometer; Sleep, sleep duration; SB, time spent in sedentary behaviour; LPA, time spent in light physical activity; MVPA, time spent in moderate-to-vigorous physical activity.

We did not find a significant difference between sleep durations estimated by DABQ and activPAL4 (mean difference (*d*) = 1.0 min/day, 95% CI: −8.7, 10.8) (Figure 1). The time spent in SB estimated by DABQ was on average 96.5 min/day less (95% CI: 124.8, 68.1) than that estimated by activPAL4. The time spent in MVPA estimated by DABQ was on average 39.1 min/day less (95% CI: 44.7, 33.5) than that estimated by activPAL4. The time spent in LPA estimated by DABQ was on average 134.5 min/day greater (95% CI: 106.8, 162.3) than the respective amounts estimated by activPAL4. The limits of agreement ranged from -98.7 to 100.8 min/day for sleep duration, from −386.5 to 193.6 min/day for SB, from −149.2 to 418.2 min/day for LPA, and from −96.3 to 18.1 min/day for MVPA. A positive proportional bias could be observed for SB estimates (*β* = 0.85, *p* < 0.001) and for LPA estimates (*β* = 1.03, *p* < 0.001).

## 4. Discussion

### 4.1. Key Findings

We found that the estimates from DABQ—a comprehensive questionnaire that enables the assessment of time spent in movement behaviours, including sleep, SB, LPA, and MVPA, across the full 24-h day—have test-retest reliability between approximately 0.60 and 0.70. The convergent validity of DABQ estimates of sleep duration tested against activPAL4 was found to be similarly high. While the convergent validity of DABQ estimates of SB, LPA, and MVPA was lower, it is comparable with the validity of most previously developed SB and physical activity questionnaires (with Spearman/Pearson correlation coefficients ranging from 0.25 to 0.41) [9,39,40]. These findings indicate that DABQ has satisfactory reliability and validity for large-scale epidemiological studies on 24-h movement behaviours.

### 4.2. Reliability and Validity of the Estimated Sleep Duration

Sleep durations estimated by DABQ have satisfactory test-retest reliability and convergent validity, which is in accordance with findings for several existing sleep questionnaires [41,42,43]. A number of previous studies established the validity of summary scores that were based on various dimensions of sleep (e.g., sleep quality, sleep disorders, daytime sleepiness) and not only on sleep duration [43,44], making direct comparisons with our findings impossible. However, some studies reported Spearman’s correlation coefficients of around 0.45 between their questionnaire estimates of sleep duration and estimates from wrist accelerometers [41,42], which is somewhat lower than the validity found in our study. DABQ may provide somewhat more valid estimates of sleep duration than these questionnaires, but their reference measures may not have been as accurate as the reference measure used in our study. While some other sleep questionnaires seem to provide a higher mean sleep duration than accelerometers [41,42], estimates of sleep duration from DABQ showed no systematic discrepancy when compared with activPAL4 estimates.

### 4.3. Reliability and Validity of the Estimated Time Spent in Sedentary Behaviour

The time spent in SB estimated by DABQ showed satisfactory test-retest reliability, comparable with the reliability of SB estimates from most existing SB questionnaires [9,39]. A recent systematic review revealed that the validity Spearman/Pearson correlation coefficients of questionnaire-based SB estimates are commonly around 0.35, similar to what we found for DABQ [9]. DABQ seems to underestimate SB to a greater extent than two out of three domain-specific SB questionnaires evaluated by Chastin et al. [21]. This may be partially because of a somewhat less detailed breakdown of SB domains in DABQ (i.e., occupational, commuting, and other non-occupational), compared with the SB questionnaire used in the Chastin et al. [21] study (i.e., work, home, transport, and leisure). However, DABQ seems to perform in this regard substantially better than, for example, the widely used IPAQ—short form and GPAQ [39].

### 4.4. Reliability and Validity of the Estimated Time Spent in Physical Activity

DABQ estimates of the time spent in LPA and MVPA showed satisfactory test-retest reliability, which is in accordance with the findings for most existing physical activity questionnaires [40]. DABQ estimates of LPA and MVPA had a similar reliability to estimates from the three most commonly used questionnaires for physical activity surveillance and monitoring in European countries (IPAQ—short form, GPAQ, and European Health Interview Survey Physical Activity Questionnaire (EHIS-PAQ)) [45]. Although the convergent validity of DABQ estimates of LPA and MVPA were somewhat lower, they were similar to the validity of physical activity estimates for most existing questionnaires. For example, the convergent validity of IPAQ estimates of MVPA against accelerometers was found to be around 0.30 [46,47] while the convergent validity of LPA estimates from a questionnaire used in the Nord-Trøndelag Health Study (HUNT) was found to be 0.21 [48]. Furthermore, estimates of MVPA from DABQ were on average significantly lower than MVPA estimates from activPAL4. We calculated the activPAL4 estimate of MVPA by using the 3 MET threshold that is based on stepping cadence, while using the events information [23]. While Lyden et al. [23] reported an excellent validity of MVPA estimated using this method, the generalisability of their findings is limited because the study was conducted in 13 participants only. It is possible that in our sample, activPAL4 overestimated MVPA. Another possible reason for this result may be in the wording of the DABQ questions. DABQ assesses occupational and other non-occupational MVPA by asking about the performance of “physically more demanding tasks” that increase breathing rate. It is possible that DABQ does not capture the entire lower-intensity spectrum of moderate physical activity in all participants because such activities might not always be perceived as physically more demanding.

### 4.5. Implications for Research and Practice

DABQ is among the first validated comprehensive questionnaires for adults that capture movement behaviours across the full 24-h day. DABQ enables assessment of sleep, SB, LPA, and MVPA on workdays and non-workdays; domain-specific SB, LPA, and MVPA; and the duration of engaging in specific types of physical activity (i.e., walking, sport participation). The importance of such detailed information on movement behaviours has been recognised, especially for the purpose of health surveillance [11,12]. The reliability and validity of DABQ for estimating the time spent in sleep, SB, LPA, and MVPA are similar to those seen in existing questionnaires that assess some of these movement behaviours, indicating that DABQ could be used for epidemiological research and health surveillance purposes. Indeed, DABQ has already been shown to be useful in a recent epidemiological study on the associations between 24-h movement behaviours and health [49].

### 4.6. Strengths and Limitations of the Study

A key strength of our study is the use of the activPAL4 accelerometer as the reference measure to evaluate the validity of DABQ estimates. A thigh-worn activPAL4 allows for relatively accurate detection of movement behaviours across the full 24-h day [23,24], which makes it a good comparison measure when evaluating the validity of movement-behaviour questionnaires. Our study had two key limitations. First, the sample included only working adults; thus, the findings might not be directly generalizable to other population groups. Second, although Spearman’s correlation coefficient, ICC, and Bland–Altman plots are widely used to evaluate the reliability and validity of movement behaviour measures [9,40], they are not intended for compositional data [7]. Given that compositional data analysis methods for evaluating the reliability and validity of 24-h movement behaviour measures are yet to be described, their conceptualisation and application were beyond the scope of this paper.

## 5. Conclusions

The newly designed DABQ enables a comprehensive assessment of movement behaviours across the full 24-h day among adults. In terms of the test-retest reliability and convergent validity of sleep, SB, LPA, and MVPA estimates, DABQ is comparable with existing questionnaires; however, it has an important advantage of enabling a comprehensive assessment of all four 24-h movement behaviours. The measurement properties of DABQ make it suitable for large-scale epidemiological studies on 24-h movement behaviours. Future studies should explore the measurement properties of DABQ in other population groups and by using compositional data analysis.

## Figures and Tables

**Table 1:** Participant characteristics (reliability sample, *n* = 114; validity sample, *n* = 107).

Characteristic	Reliability Sample *n* (%)	Validity Sample *n* (%)
Age group		
24 to 34 years	42 (36.8)	38 (35.5)
35 to 45 years	46 (40.4)	44 (41.1)
46 to 56 years	26 (22.8)	25 (23.4)
Sex		
Female	46 (40.4)	45 (42.1)
Male	68 (59.6)	62 (57.9)
BMI category		
“Normal” weight (18.0 to 24.9 kg/m^2^)	53 (46.5)	52 (48.6)
Overweight (25.0 to 29.9 kg/m^2^)	39 (34.2)	35 (32.7)
Obese (≥ 30 kg/m^2^)	22 (19.3)	20 (18.7)
Education		
Primary or secondary education	27 (23.7)	22 (20.6)
Tertiary education (college)	30 (26.3)	29 (27.1)
Tertiary education (university)	57 (50.0)	56 (52.3)
Work schedule		
Non-shift work	58 (50.9)	55 (51.4)
Shift work	56 (49.1)	52 (48.6)

Note: BMI, body mass index.

**Table 2:** Test-retest reliability of the Daily Activity Behaviours Questionnaire (DABQ).

Movement Behaviour	DABQ1 Mean (SD), min/day	DABQ2 Mean (SD), min/day	ICC[A,1] (95% CI)	ICC[C,1] (95% CI)	Spearman’s *ρ* (95% CI)
Sleep	430 (54)	443 (63)	0.59 (0.48, 0.69)	0.61 (0.50, 0.69)	0.64 (0.52, 0.74)
SB	547 (163)	547 (166)	0.65 (0.55, 0.73)	0.65 (0.55, 0.73)	0.62 (0.49, 0.72)
LPA	423 (165)	416 (167)	0.69 (0.60, 0.77)	0.69 (0.60, 0.77)	0.67 (0.56, 0.76)
MVPA	40 (41)	34 (35)	0.65 (0.55, 0.73)	0.65 (0.56, 0.74)	0.60 (0.47, 0.71)

Abbreviations: DABQ1, Daily Activity Behaviours Questionnaire completed on the first occasion; DABQ2, Daily Activity Behaviours Questionnaire completed on the second occasion; Sleep, sleep duration; SB, time spent in sedentary behaviour; LPA, time spent in light physical activity; MVPA, time spent in moderate-to-vigorous physical activity.

**Table 3:** Convergent validity of the Daily Activity Behaviours Questionnaire (DABQ) compared against activPAL4.

Movement Behaviour	DABQ2 Mean (SD), min/day	activPAL4 Mean (SD), min/day	ICC[A,1] (95% CI)	ICC[C,1] (95% CI)	Spearman’s *ρ* (95% CI)
Sleep	443 (64)	442 (52)	0.63 (0.52, 0.71)	0.63 (0.52, 0.71)	0.66 (0.54, 0.76)
SB	547 (165)	643 (85)	0.29 (0.09, 0.45)	0.36 (0.22, 0.49)	0.42 (0.25, 0.56)
LPA	418 (165)	284 (70)	0.22 (−0.00, 0.41)	0.35 (0.20, 0.48)	0.45 (0.29, 0.59)
MVPA	31 (32)	71 (25)	0.24 (−0.05, 0.48)	0.47 (0.33, 0.58)	0.38 (0.21, 0.53)

Abbreviations: DABQ2, Daily Activity Behaviours Questionnaire completed on the second occasion; activPAL4, activPAL4 accelerometer/inclinometer; Sleep, sleep duration; SB, time spent in sedentary behaviour; LPA, time spent in light physical activity; MVPA, time spent in moderate-to-vigorous physical activity.

## Data Availability

The data presented in this study are available on request from the corresponding author.

## Supplementary Materials

The following supporting information can be downloaded at: https://www.mdpi.com/article/10.3390/ijerph19095362/s1, Table S1: Test-retest reliability of the Daily Activity Behaviours Questionnaire (DABQ)—extended list.
