# Supplementary material for: Validity and Reliability of the Daily Activity Behaviours Questionnaire (DABQ) for Assessment of Time Spent in Sleep, Sedentary Behaviour, and Physical Activity
Source: Int J Environ Res Public Health. 2022 Apr 28;19(9):5362. doi: 10.3390/ijerph19095362 (PMC9100796; doi:10.3390/ijerph19095362)
Supplement: Supplementary file 1 [file ijerph-19-05362-s001.zip › ijerph-1664408-supplementary.pdf]

Table S1. Test-retest reliability of the Daily Activity Behaviours Questionnaire (DABQ)—extended list.

Movement Behaviour	DABQ1 Mean (SD), min/day	DABQ2 Mean (SD), min/day	ICC[A,1] (95% CI)	ICC[C,1] (95% CI)	Spearman's ρ
**Time in bed**	453 (51)	462 (62)	0.63 (0.53, 0.71)	0.64 (0.53, 0.72)	0.67 (0.56, 0.76)
Sleep latency	18 (14)	17 (12)	0.67 (0.57, 0.74)	0.67 (0.58, 0.75)	0.79 (0.71, 0.85)
Wake after sleep onset	5 (12)	3 (6)	0.42 (0.29, 0.54)	0.44 (0.30, 0.55)	0.51 (0.36, 0.63)
Napping time	6 (9)	9 (12)	0.32 (0.18, 0.46)	0.33 (0.19, 0.46)	0.43 (0.27, 0.57)
**Occupational time**	559 (120)	552 (110)	0.77 (0.70, 0.83)	0.77 (0.70, 0.83)	0.74 (0.64, 0.81)
Occupational SB	396 (129)	362 (122)	0.66 (0.55, 0.75)	0.68 (0.59, 0.76)	0.70 (0.60, 0.79)
Occupational PA	163 (118)	190 (125)	0.72 (0.63, 0.79)	0.73 (0.65, 0.80)	0.73 (0.63, 0.81)
**Commuting time**	60 (66)	52 (61)	0.91 (0.87, 0.93)	0.91 (0.88, 0.93)	0.87 (0.81, 0.91)
SB while commuting	56 (68)	48 (63)	0.91 (0.88, 0.93)	0.92 (0.89, 0.94)	0.90 (0.85, 0.93)
PA while commuting	4 (10)	4 (10)	0.88 (0.84, 0.91)	0.88 (0.84, 0.91)	0.92 (0.89, 0.95)
**Non-occupational time**[1]	430 (131)	433 (127)	0.75 (0.67, 0.81)	0.75 (0.67, 0.81)	0.73 (0.63, 0.81)
Non-occupational SB	177 (119)	207 (121)	0.49 (0.36, 0.60)	0.50 (0.38, 0.61)	0.47 (0.31, 0.60)
Non-occupational PA	253 (141)	226 (134)	0.59 (0.47, 0.68)	0.60 (0.49, 0.69)	0.60 (0.67, 0.71)
**Workday**					
Sleep on workday	391 (72)	404 (77)	0.62 (0.51, 0.71)	0.63 (0.53, 0.71)	0.66 (0.54, 0.75)
SB on workday	628 (190)	616 (186)	0.62 (0.51, 0.71)	0.62 (0.51, 0.71)	0.57 (0.43, 0.68)
PA on workday	421 (194)	419 (191)	0.69 (0.60, 0.77)	0.69 (0.60, 0.77)	0.67 (0.56, 0.76)
**Non-workday**					
Sleep on non-workday	498 (80)	497 (93)	0.53 (0.41, 0.63)	0.53 (0.41, 0.63)	0.69 (0.58, 0.78)
SB on non-workday	391 (184)	429 (195)	0.48 (0.35, 0.59)	0.48 (0.36, 0.59)	0.46 (0.30, 0.59)
PA on non-workday	551 (199)	501 (194)	0.59 (0.47, 0.68)	0.60 (0.50, 0.69)	0.57 (0.43, 0.68)
**Non-occupational PA**					
Walking	46 (40)	44 (33)	0.37 (0.23, 0.49)	0.37 (0.23, 0.49)	0.47 (0.31, 0.60)
Sport participation	21 (27)	21 (30)	0.62 (0.52, 0.71)	0.62 (0.52, 0.71)	0.71 (0.61, 0.79)
Other demanding activity	14 (23)	12 (22)	0.36 (0.22, 0.49)	0.36 (0.22, 0.49)	0.50 (0.35, 0.63)

Abbreviations: DABQ1, Daily Activity Behaviours Questionnaire completed on the first occasion; DABQ2, Daily Activity Behaviours Questionnaire completed on the second occasion; Sleep, sleep duration; SB, time spent in sedentary behaviour; PA, time spent in physical activity; MSE, muscle-strengthening exercise; [1] non-occupational time on workday, excluding commuting time
